# Postoperative elevated bed header position versus supine in the management of chronic subdural hematoma: a systematic review and meta-analysis

**DOI:** 10.1007/s13760-024-02571-4

**Published:** 2024-05-27

**Authors:** Ibrahim Serag, Mariam Abdelhady, Abdelaziz A. Awad, Ahmed Wageeh, Ahmed Shaboub, Rowan H. Elhalag, Ahmed Aljabali, Mohamed Abouzid

**Affiliations:** 1 Faculty of Medicine, Mansoura University, Mansoura, Egypt; 2 Faculty of Medicine, October 6 University, Giza, Egypt; 3 Faculty of Medicine, Azhar University, Cairo, Egypt; 4 Faculty of Medicine, Menoufia University, Menoufia, Egypt; 5 Faculty of Medicine, Ain Shams University, Cairo, Egypt; 6 Faculty of Medicine, Alexandria University, Alexandria, Egypt; 7 Faculty of Medicine, Jordan University of Science and Technology, Irbid, Jordan; 8 Department of Physical Pharmacy and Pharmacokinetics, Faculty of Pharmacy, Poznan University of Medical Sciences, Rokietnicka 3 St, 60-806 Poznan, Poland; 9 Doctoral School, Poznan University of Medical Sciences, 60-812 Poznan, Poland

**Keywords:** Chronic subdural hematoma, Elevated head position, Supine head position

## Abstract

**Background:**

Chronic subdural hematoma (CSDH) is a prevalent type of intracranial hemorrhage. Surgical interventions, such as Twist Drill Craniostomy and Burr Hole Craniostomy, are employed for its treatment. However, limited information exists regarding the impact of postoperative head position (supine vs. elevated) on clinical outcomes. We aim to assess whether patients’ head position after surgery influences their prognosis.

**Method:**

We conducted a PRISMA-compliant systematic review and meta-analysis. Our search encompassed PubMed, Cochrane CENTRAL, Scopus, Web of Science, and Embase databases to identify relevant published studies. Data were meticulously extracted, pooled using a fixed model, and reported as risk ratios (RR) with 95% confidence intervals (CI). Statistical analysis was performed using R and Stata MP v.17.

**Results:**

Five studies involving 284 patients were included in our meta-analysis. We focused on three primary clinical outcomes, comparing the supine and elevated header positions. Notably, there was no statistically significant difference between the supine and elevated positions in terms of recurrence rate (RR 0.77, 95% CI [0.44, 1.37]), second intervention for recurrence (RR 1.07, 95% CI [0.42, 2.78]) and postoperative complications (RR 1.16, 95% CI [0.70, 1.92]).

**Conclusion:**

Current studies have proved no difference between supine and elevated bed header positions regarding recurrence rate, second intervention for recurrence, and postoperative complications. Future RCTs with long-term follow-ups are recommended.

## Introduction

Chronic subdural hematoma (CSDH) is a common intracranial hemorrhage seen predominantly in older adults. As the population ages and anticoagulant medication usage becomes more widespread, the incidence of CSDH is on the rise [1, 2]. The annual occurrence of CSDH in the adult population varies from 1.7 to 16.3 cases per 100,000 individuals [3, 4]. Clinically, CSDH is characterized by a collection of blood or its breakdown products between the brain and the dura mater, persisting for at least 21 days [5]. Recurrence rates range from 9.2 to 26.5% [6–11].

Management strategies for CSDH include various surgical approaches, such as twist drill craniostomy with closed system drainage (TDC-CSD) and Burr Hole Craniostomy [4]. While these interventions have demonstrated long-standing success, there remains limited knowledge regarding the impact of head position (flat/supine vs. elevated/reverse Trendelenburg) on procedural efficacy and overall patient outcomes.

Craniostomy, a common surgical procedure for CSDH, is typically performed with the patient in a semi-lateral or supine position [12]. The head can be kept neutral or rotated, depending on the location of the lesion [12]. During this procedure, it is crucial to position the burr hole near the vertex of the head. Improper head posture during burr hole creation may lead to the accumulation of significant subdural air [12, 13].

CSDH often occurs alongside cerebral atrophy, increasing potential space within the subdural area. To facilitate drainage and reduce this potential space, some neurosurgeons advocate flattening the patient’s head and adjusting the head end of the bed. However, an alternative approach involves elevating the head end of the bed. This technique, borrowed from the management of acute subdural hematomas, aims to decrease intracranial pressure and is also applied to CSDH patients [14].

The growth of CSDH involves complex mechanisms that continue to be debated. Two main hypotheses shed light on its pathogenesis: (i) Oncotic Pressure and Clot Liquefaction: Some researchers propose that increasing oncotic pressure within the enclosed region due to partial clot liquefaction contributes to CSDH progression. While this concept remains controversial, adjusting the patient’s head end of the bed may help modulate the pressure gradient [15]. (ii) Recurrent Bleeding and Aberrant Veins: Another hypothesis attributes CSDH spread to repeated bleeding. Dilated, aberrant veins within the hematoma’s outer layer are implicated in this process [16]. Exudation from macro capillaries in the CSDH outer membrane likely plays a crucial role in disease progression. Elevating the head end of the bed during treatment may mitigate this source of hemorrhage [17].

During surgery, particularly in awake patients, the sitting position offers relaxation and convenience for both the patient and the surgeon. The burr hole, created near the patient’s vertex (and the vertex of the CSDH), acts as a natural barrier, preventing gas entry into the subdural cavity. Although the sitting position has historically been associated with complications (such as venous air embolism), recent studies show reduced complication rates in patients undergoing surgeries in this position [18, 19].

Furthermore, some authors advocate early post-procedure head elevation and prompt mobilization to prevent complications related to prolonged recumbency, including atelectasis, pneumonia, and venous thromboembolic diseases [20, 21]. However, anesthesiologists and surgeons approach this position cautiously [22].

While our previous work showed that various factors have been associated with the recurrence of CSDH and other outcomes [23–26], optimal patient positioning remains uncertain. This study aims to provide updated evidence and compare different bedside positions regarding their impact on recurrences and complications. By examining these factors, we hope to enhance our understanding of effective management strategies for CSDH.

## Methods

### Literature search

We conducted a thorough literature review across various databases, including PubMed, Cochrane CENTRAL, Scopus, Web of Science, and Ovid, with the latest search performed in August 2023. Our search strategy utilized Medical Subject Headings terms, including ("subdural hematoma" or "chronic subdural hematoma") in combination with ("supine" or "elevated" or "bed header"). To ensure inclusivity, we performed the search without any filters. Additionally, we adhered to a meticulous approach by manually scrutinizing the reference lists of studies meeting our initial criteria, ensuring that no potentially relevant studies were inadvertently omitted during our search process.

### Eligibility criteria

We followed a defined set of criteria based on the PICO framework: (1) Patients with CSDH; (2) Elevated head position; (3) Supin head position; (4) Assessment of morbidity, mortality, and recurrence rates. Our selection criteria excluded studies that did not meet the PICO criteria, as well as case reports, review papers, and secondary reviews. Additionally, conference abstracts were excluded due to their tendency to present incomplete or insufficiently detailed results.

### Study selection and data extraction

Two reviewers (IS and AW) initially screened studies based on titles and abstracts using Rayyan [27]. Subsequently, the complete texts of potentially eligible studies underwent comprehensive evaluation for final inclusion. Disagreements between reviewers prompted constructive discussions to reach consensus, with a third reviewer (AA) making the final decision if consensus was unattainable. For included studies, two authors (IS and AS) independently extracted data using standardized templates on Google Sheets for outcomes and study population characteristics, including study ID, country, study design, sample size, follow-up period in months, gender distribution, mean age at baseline, inclusion criteria and exclusion criteria for each study.

### Quality assessment

Two reviewers independently evaluated the quality of the included studies (IS and AW), and a third author resolved any disagreements (MAzid). For observational studies, we employed a modified New Castle Ottawa scale consisting of eight questions distributed across the domains of selection (four questions), comparability (one question), and outcome (three questions). Each question carried a maximum score of 1 point, except for comparability, which could be assigned 2 points [28]. Ratings were categorized as "high quality" (7–9 points), "some concern" (5–6 points), and "low quality" (0–4 points).

Randomized Controlled Trials (RCTs) underwent assessment using the Cochrane risk of bias tool 2, with each RCT categorized as high quality, some concern, or low quality [29].

### Data synthesis and statistical analysis

If sufficient studies addressing a specific outcome were available, we conducted a meta-analysis using R and StataMP version 17. The Mantel–Haenszel method used the fixed-effect model to calculate the summary estimate. Dichotomous data were computed using risk ratios (RR) and 95% confidence interval (CI), and continuous data were calculated using mean and standard deviation. The chi-square statistic was used to calculate I-squared. A Chi-square with a p-value less than 0.1 was considered significant heterogeneity. Also, the I-square value of more than or equal to 50% indicated high heterogeneity [30]. Also, we performed meta-regression with each outcome, using the average age of the patients in each study, and the results were reported as standard error (SE) and 95% CI. We conducted a sensitivity analysis to evaluate the impact of these studies on the overall assessment of results.

## Results

### Study selection

**Fig. 1:**
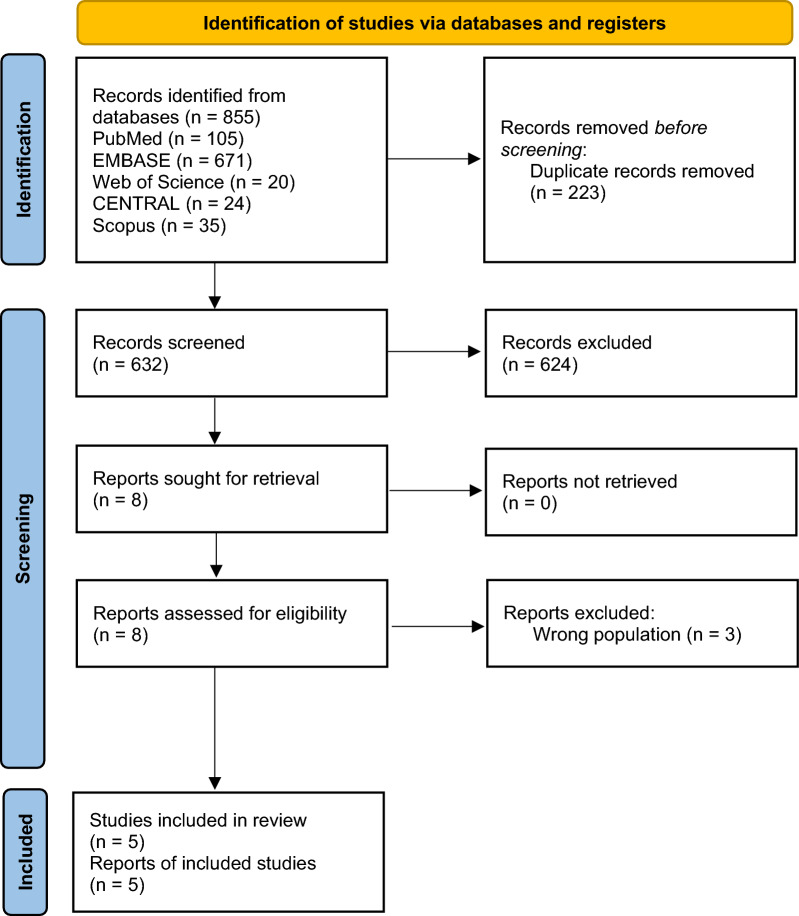
PRISMA chart of the reported studies showing the search selection strategy and exclusion criteria [34]

Our investigation yielded a comprehensive collection of 855 articles, sourced as follows: 105 from Pubmed, 20 from WOS, 671 from Embase, 24 from Cochrane, and 35 from Scopus. After eliminating 223 duplicates, we conducted title and abstract screenings on eight articles, excluding an additional three. Subsequent full-text screening retained five articles for quantitative analysis [3, 4, 14, 31, 32] (refer to PRISMA Fig. 1).

### Quality, summary, and baseline characteristics of included studies

**Fig. 2:**
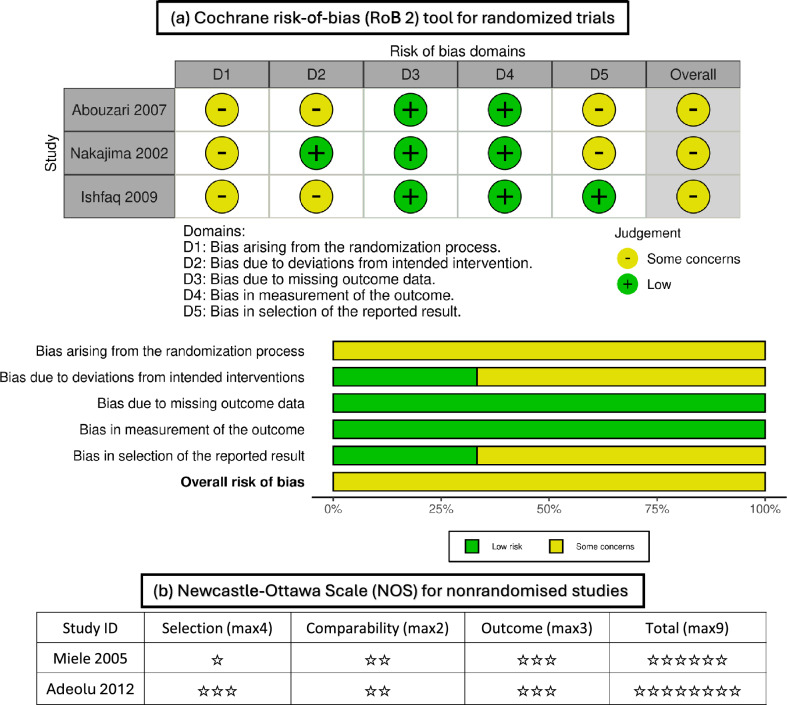
Risk of bias assessment is represented in traffic light plot and summary plot according to the Cochrane risk-of-bias tool, created using robvis [35] and The Newcastle–Ottawa Scale assessment for nonrandomized studies

The quality assessment of the included studies, as determined by the Cochrane risk of bias assessment, indicated a moderate to high quality. Figure 2 provides a comprehensive overview of the quality assessment domains for three studies, showing that all three RCTs had some concerns in the randomization process; some concerns were also found in some studies regarding selection and deviations from the intended intervention. Non-RCT trials showed that the study of Adeolu et al. [26] was of high quality, and some concerns in selection were found in Miele et al. [4].

For the summary of the studies, there were 146 patients in the study group and 138 patients in the control group, of which we included 284 patients. Table 1 briefly summarizes baseline characteristics and main findings of the included studies.

**Table 1:** Baseline characteristics and main findings of the included studies

Study and year	Study design	Country	Sample size			Age (mean, SD)		Sex (male number, %)		Inclusion criteria	Exclusion criteria	Main findings
			Supine	Elevated	total	Supine	Elevated	Supine	Elevated			
Miele 2005 [4]	Retrospective cohort	USA (West Virginia)	24	20	44	N/A	N/A	N/A	N/A	(1) Patients were required to have a CT scan demonstrating a hypodense or isodense collection in the subdural space (2) The patient had to be symptomatic to be included in the study	N/A	Elevation of the patient’s head during TDC-CSD treatment of CSDH does not seem to Impact the amount of drainage, recurrence frequency, or complication rate. A statistically significant difference in length of stay in the hospital was observed
Ishfaq 2009 [14]	non-RCT	Pakistan	30	30	60	61.9 (14.4)	58.1 (12.9)	46 (77%)		N/A	N/A	A 30-degree head-up position soon after operation in cases of CSDH does not significantly affect the outcome and recurrence
Abouzari 2007 [3]	RCT	Tehran	42	42	84	57.8 (8.1)	59.4 (13.7)	30 (71%)	29 (69%)	(1) The presence of a typical neomembrane (2) Typical liquefied blood within the hematoma cavity (3) If at least three weeks have passed since the head trauma event	In the study, patients without a clear history of trauma were included. However, those with one or more documented risk factors that could influence recurrence were excluded. Also, hygroma, calcified or ossified CSDH, and asymptomatic cases were excluded from the analysis	Following burr-hole surgery, assuming an upright posture was significantly linked to an increased incidence of CSDH recurrence. However, this positioning did not alter substantially other complications related to the surgical procedure
Nakajima 2002 [32]	RCT	Japan	25	21	46	71	74.9	17 (68%)	15 (71%)	Patients with CSDH	N/A	An upright posture soon after the operation in CSDH cases is not considered a risk factor for recurrence
Adeolu 2012 [31]	Prospective cohort study	Nigeria	25	25	50	57 (15.04)		23 (92%)	20 (80%)	Patients with symptomatic subacute/CSDH confirmed by CT scans underwent burr-hole drainage of the hematoma	Patients who could not mobilize in the early postoperative period	Both early and late mobilization (sitting vs. spine) groups were equally beneficial in the postoperative care of patients following burr-hole drainage of subacute/CSDH

CSDH: chronic subdural hematomas; CT: computed tomography; RCT: randomized controlled trial; SD: standard deviation; TDC-CSD: twist drill craniostomy with closed system drainage; N/A: not applicable

### Outcomes

**Fig. 3:**
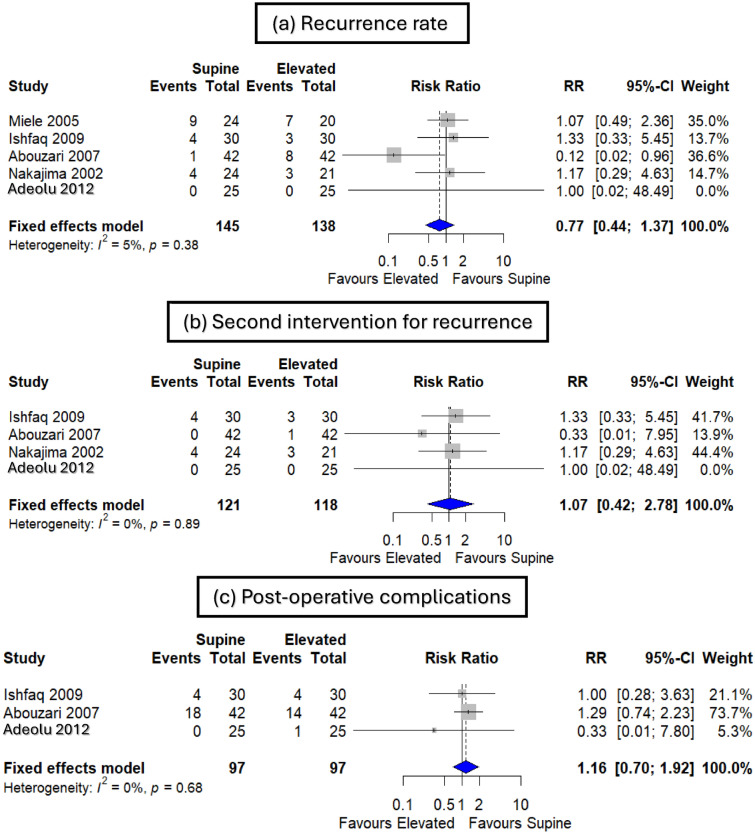
Forest plot of (**a**) recurrence rate, (**b**) second intervention for recurrence, and (**c**) postoperative complications

Supine and elevated bed header positions showed no statistically significant difference in recurrence rate (RR 0.77 and 95% CI [0.44, 1.37], p = 0.38), second intervention for recurrence (RR 1.07 and 95% CI [0.42, 2.78], p = 0.89), and postoperative complications (RR 1.16 and 95% CI [0.70, 1.92], p = 0.68) (Fig. 3a–c). There was no significant heterogeneity observed in recurrence rate (p = 0.38, I^2^ = 5%), second intervention for recurrence (p = 0.89, I^2^ = 0%), and postoperative complications (p = 0.68, I^2^ = 0%) (Fig. 3a–c).

### Meta-regression

We did not observe any significant impact of age among the three outcomes: recurrence rate (SE 0.066 and 95% CI [− 0.078, 0.180]), second intervention for recurrence (SE 0.011 and 95% CI [− 0.126, 0.147]) and postoperative complications (SE 0.444 and 95% CI [− 0.829, 0.912]) (Table 2).

**Table 2:** Meta-regression performed with age as moderator

Outcome	Coefficient	Std. err	z	p >|z|	− 95% CI	+ 95% CI
Recurrence rate	0.051	0.066	0.770	0.440	− 0.078	0.180
Second intervention for recurrence	0.011	0.069	0.150	0.879	− 0.126	0.147
Post-operative complications	0.042	0.444	0.090	0.926	− 0.829	0.912

### Sensitivity analysis.

**Fig. 4:**
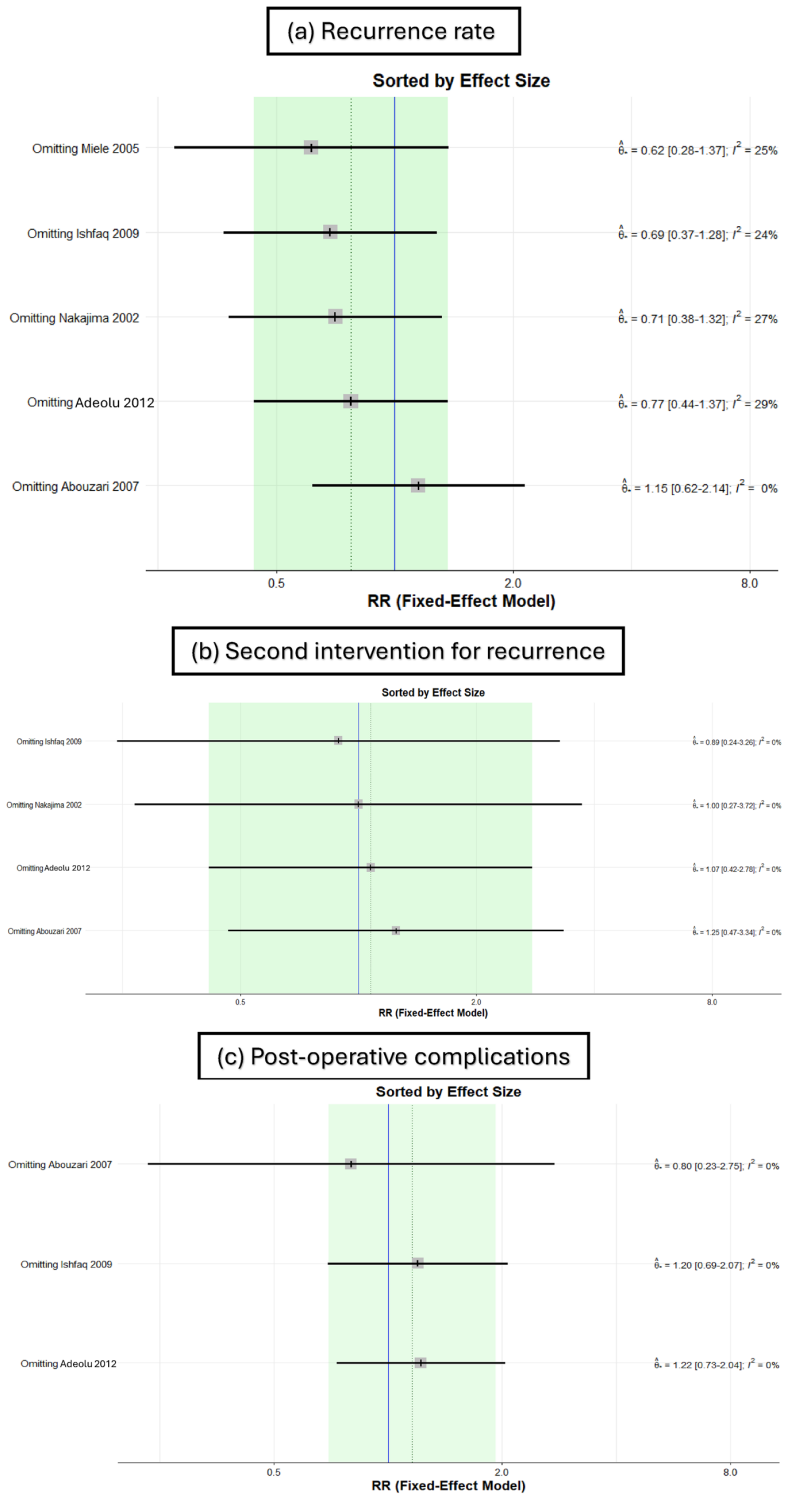
Senstivity analysis of (**a**) recurrence rate, (**b**) second intervention for recurrence, and (**c**) postoperative complications

The sensitivity analysis consistently maintained the same results for all outcomes. The I^2^ statistic ranged from 0 to 29%, indicating low heterogeneity in the data, thus suggesting a high level of homogeneity (Fig. 4a–c).

## Discussion

The systematic review and meta-analysis on the impact of postoperative bed header position (elevated vs. supine) in managing CSDH revealed no statistically significant differences in clinical outcomes between the two positioning strategies. The study pooled data from five studies, encompassing 284 patients, and discussed three primary clinical outcomes: recurrence rate, the second intervention due to recurrence, and postoperative complications. The findings, expressed as risk ratios and confidence intervals, indicated that the elevation of the bed header post-operatively did not significantly influence the recurrence, the need for second interventions, or the incidence of postoperative complications compared to the supine position.

These findings suggest that the position of the bed header, whether elevated or supine, post-CSDH surgery does not have a differential impact on the recurrence rates, requirement for additional surgical interventions, or the development of complications. This outcome underscores the need for further research, particularly randomized controlled trials with long-term follow-ups, to explore other factors influencing postoperative outcomes in CSDH management.

In comparing our findings with those of a previous meta-analysis conducted in 2014 [33], both studies corroborate the conclusion that there is no statistically significant difference in recurrence rates, necessity for reoperation, or postoperative complications between elevated and supine bed header positions following surgery for CSDH. The earlier meta-analysis reported a slightly suggestive but non-significant trend towards reduced recurrences with an elevated bed header, with relative risks closely aligning with those found in our study (Recurrence: 0.51 vs. our RR = 0.77; Reoperation: 1.07 vs. our RR = 1. 07; Complications: 1.15 vs. our RR = 1.16). Both analyses underscore the critical need for further high-quality randomized controlled trials to increase the quality of evidence of the impact of postoperative positioning on CSDH management outcomes.

The significance of the postoperative bed header position in managing CSDH has been debated considerably, with varying practices and outcomes reported in the literature. Our findings align with those of Adeolu et al. [31] and Ishfaq et al. [14], indicating that the elevation of the bed header post-operatively does not significantly affect the rate of hematoma recurrence or the incidence of postoperative complications [14, 31]. This suggests that early mobilization, regardless of bed header position, may be a viable option for patients, potentially facilitating quicker recovery without increasing risks. Contrarily, Abouzari et al. [3] reported an increased recurrence rate associated with an upright posture soon after surgery, highlighting the potential risk factors that must be considered when deciding postoperative care [3]. Despite these differing outcomes, our manuscript, along with the study by Ishfaq et al. [14], suggests that the positioning of the patient's head, either flat or elevated, does not grant access to the operculum or significantly influence the surgical efficacy in terms of hematoma evacuation. Furthermore, the study by Adeolu et al. [31] underscores the absence of significant differences in outcomes between early and late mobilization, advocating for the benefits of early patient mobilization to reduce hospital stays without exacerbating side effects or recurrence rates.

## Limitations

First, including non-RCTs alongside RCTs while expanding the scope of the analysis introduces a potential bias that could skew the results. Non-RCTs are more susceptible to confounding factors and selection bias, which might affect the validity of the comparisons made between different pharmacological interventions. Second, although the sample size provided robust results based on the sensitivity analysis, it was not sufficiently large enough to conduct subgroup analyses with higher statistical power. Third, our study did not account for all potential variables, such as variations in surgical technique, the exact volume of hematoma drainage, or patients' comorbid conditions, which could affect outcomes independently of bed header position. Finally, the follow-up period may not have been long enough to capture late recurrences or long-term complications.

## Conclusion

No substantial disparity exists between the elevated and supine bed header positions concerning recurrence rates, second interventions for recurrence, or postoperative complications. However, we recommend conducting future randomized controlled trials with larger patients and long-term follow-ups to further elucidate optimal management strategies for CSDH patients.

## Data Availability

All data generated or analyzed during this study are included in this published article.

## References

[1] Rauhala M, Luoto TM, Huhtala H, Iverson GL, Niskakangas T, Öhman J et al (2019) The incidence of chronic subdural hematomas from 1990 to 2015 in a defined Finnish population. J Neurosurg 132:1147–1157. 10.3171/2018.12.JNS183035 30901751 10.3171/2018.12.JNS183035

[2] Ducruet AF, Grobelny BT, Zacharia BE, Hickman ZL, DeRosa PL, Andersen KN et al (2012) The surgical management of chronic subdural hematoma. Neurosurg Rev 35:155–169. 10.1007/s10143-011-0349-y 21909694 10.1007/s10143-011-0349-y

[3] Abouzari M, Rashidi A, Rezaii J, Esfandiari K, Asadollahi M, Aleali H et al (2007) The role of postoperative patient posture in the recurrence of traumatic chronic subdural hematoma after burr-hole surgery. Neurosurgery 61:794–797. 10.1227/01.NEU.0000298908.94129.67 17986941 10.1227/01.NEU.0000298908.94129.67

[4] Miele VJ, Sadrolhefazi A, Bailes JE (2005) Influence of head position on the effectiveness of twist drill craniostomy for chronic subdural hematoma. Surg Neurol 63:420–423. 10.1016/j.surneu.2004.06.023 15883061 10.1016/j.surneu.2004.06.023

[5] Macro V, Alexander W. Traumatic Intracranial Haematomas. Setti SR Richard GE Eds, Principles of Neurosurgery; 2005, p. 363–4.

[6] Wakai S, Hashimoto K, Watanabe N, Inoh S, Ochiai C, Nagai M (1990) Efficacy of closed-system drainage in treating chronic subdural hematoma: a prospective comparative study. Neurosurgery 26:771–773. 10.1097/00006123-199005000-00006 2352594 10.1097/00006123-199005000-00006

[7] El-Kadi H, Miele VJ, Kaufman HH (2000) Prognosis of chronic subdural hematomas. Neurosurg Clin N Am 11:553–567 10918029 10.1016/S1042-3680(18)30122-0

[8] Ernestus RI, Beldzinski P, Lanfermann H, Klug N (1997) Chronic subdural hematoma: surgical treatment and outcome in 104 patients. Surg Neurol 48:220–225. 10.1016/s0090-3019(97)80031-6 9290707 10.1016/s0090-3019(97)80031-6

[9] Fukuhara T, Gotoh M, Asari S, Ohmoto T, Akioka T (1996) The relationship between brain surface elastance and brain reexpansion after evacuation of chronic subdural hematoma. Surg Neurol 45:570–574. 10.1016/0090-3019(95)00471-8 8638244 10.1016/0090-3019(95)00471-8

[10] Kotwica Z, Brzeziński J (1991) Chronic subdural haematoma treated by burr holes and closed system drainage: personal experience in 131 patients. Br J Neurosurg 5:461–465. 10.3109/02688699108998474 1764227 10.3109/02688699108998474

[11] Nakaguchi H, Tanishima T, Yoshimasu N (2001) Factors in the natural history of chronic subdural hematomas that influence their postoperative recurrence. J Neurosurg 95:256–262. 10.3171/jns.2001.95.2.0256 11780895 10.3171/jns.2001.95.2.0256

[12] Winn HR. Youmans and Winn Neurological Surgery E-Book: 4-Volume Set. Elsevier Health Sciences; 2022.

[13] You C, Zheng X (2018) Postoperative pneumocephalus increases the recurrence rate of chronic subdural hematoma. Clin Neurol Neurosurg 166:56–60. 10.1016/j.clineuro.2018.01.029 29408774 10.1016/j.clineuro.2018.01.029

[14] Ishfaq A, Ahmed I, Bhatti SH (2009) Effect of head positioning on outcome after burr hole craniostomy for chronic subdural haematoma. J Coll Physicians Surg-Pak JCPSP 19:492–495 19651011

[15] Williams GR, Baskaya MK, Menendez J, Polin R, Willis B, Nanda A (2001) Burr-hole versus twist-drill drainage for the evacuation of chronic subdural haematoma: a comparison of clinical results. J Clin Neurosci Off J Neurosurg Soc Australas 8:551–554. 10.1054/jocn.2000.0926 10.1054/jocn.2000.0926 11683603

[16] Stoodley M, Weir B (2000) Contents of chronic subdural hematoma. Neurosurg Clin N Am 11:425–434 10918011 10.1016/S1042-3680(18)30104-9

[17] Tokmak M, Iplikcioglu AC, Bek S, Gökduman CA, Erdal M (2007) The role of exudation in chronic subdural hematomas. J Neurosurg 107:290–295. 10.3171/JNS-07/08/0290 17695382 10.3171/JNS-07/08/0290

[18] Ammirati M, Lamki TT, Shaw AB, Forde B, Nakano I, Mani M (2013) A streamlined protocol for the use of the semi-sitting position in neurosurgery: a report on 48 consecutive procedures. J Clin Neurosci Off J Neurosurg Soc Australas 20:32–34. 10.1016/j.jocn.2012.05.037 10.1016/j.jocn.2012.05.037 PMC3840951 23178073

[19] Himes BT, Mallory GW, Abcejo AS, Pasternak J, Atkinson JLD, Meyer FB et al (2017) Contemporary analysis of the intraoperative and perioperative complications of neurosurgical procedures performed in the sitting position. J Neurosurg 127:182–188. 10.3171/2016.5.JNS152328 27494821 10.3171/2016.5.JNS152328

[20] Perme C, Chandrashekar R (2009) Early mobility and walking program for patients in intensive care units: creating a standard of care. Am J Crit Care Off Publ Am Assoc Crit-Care Nurses 18:212–221. 10.4037/ajcc2009598 10.4037/ajcc2009598 19234100

[21] Kurabe S, Ozawa T, Watanabe T, Aiba T (2010) Efficacy and safety of postoperative early mobilization for chronic subdural hematoma in elderly patients. Acta Neurochir 152:1171–1174. 10.1007/s00701-010-0627-4 20336332 10.1007/s00701-010-0627-4

[22] Jadik S, Wissing H, Friedrich K, Beck J, Seifert V, Raabe A (2009) A standardized protocol for the prevention of clinically relevant venous air embolism during neurosurgical interventions in the semisitting position. Neurosurgery 64:533–538. 10.1227/01.NEU.0000338432.55235.D3 19240616 10.1227/01.NEU.0000338432.55235.D3

[23] Abdelhady MA, Aljabali A, Al-Jafari M, Serag I, Elrosasy A, Atia A et al (2024) Local anesthesia with sedation and general anesthesia for the treatment of chronic subdural hematoma: a systematic review and meta-analysis. Neurosurg Rev 47:162. 10.1007/s10143-024-02420-1 38627254 10.1007/s10143-024-02420-1 PMC11021259

[24] Alkhawaldeh I, Serag I, Abouzid M, Hamdallah A, Al-Jafari M, Abdelhady M et al (2024) A comparison of subperiosteal or subgaleal drainage with subdural drainage on the outcome of chronic subdural hematoma surgery (P7–11012). Neurology 102:3247. 10.1212/WNL.0000000000205015 10.1212/WNL.0000000000205015

[25] Aljabali A, Sharkawy AM, Jaradat B, Serag I, Al-dardery NM, Abdelhady M et al (2023) Drainage versus no drainage after burr-hole evacuation of chronic subdural hematoma: a systematic review and meta-analysis of 1961 patients. Neurosurg Rev 46:251. 10.1007/s10143-023-02153-7 37726502 10.1007/s10143-023-02153-7 PMC10509130

[26] Aljabali A, Serag I, Diab D, Alhadeethi A, Abdelhady M, Alkhawaldeh I et al (2024) Irrigation versus no irrigation in the treatment of chronic subdural hematoma: an updated systematic review and meta-analysis of 1581 patients. Neurosurg Rev. 10.1007/s10143-024-02368-2 38538863 10.1007/s10143-024-02368-2

[27] Ouzzani M, Hammady H, Fedorowicz Z, Elmagarmid A (2016) Rayyan-a web and mobile app for systematic reviews. Syst Rev 5:210. 10.1186/s13643-016-0384-4 27919275 10.1186/s13643-016-0384-4 PMC5139140

[28] Stang A (2010) Critical evaluation of the Newcastle-Ottawa scale for the assessment of the quality of nonrandomized studies in meta-analyses. Eur J Epidemiol 25:603–605. 10.1007/s10654-010-9491-z 20652370 10.1007/s10654-010-9491-z

[29] Sterne JAC, Savović J, Page MJ, Elbers RG, Blencowe NS, Boutron I et al (2019) RoB 2: a revised tool for assessing risk of bias in randomised trials. BMJ 366:l4898. 10.1136/bmj.l4898 31462531 10.1136/bmj.l4898

[30] Joanne E McKenzie, Sue E Brennan, Rebecca E Ryan, Hilary J Thomson, Renea V Johnston. Chapter 9: Summarizing study characteristics and preparing for synthesis. Cochrane Handb. Syst. Rev. Interv., Cochrane; 2023.

[31] Adeolu AA, Rabiu TB, Adeleye AO (2012) Post-operative day two versus day seven mobilization after burr-hole drainage of subacute and chronic subdural haematoma in Nigerians. Br J Neurosurg 26:743–746. 10.3109/02688697.2012.690912 22905886 10.3109/02688697.2012.690912

[32] Nakajima H, Yasui T, Nishikawa M, Kishi H, Kan M (2002) The role of postoperative patient posture in the recurrence of chronic subdural hematoma: a prospective randomized trial. Surg Neurol 58:385–387. 10.1016/S0090-3019(02)00921-7 12517615 10.1016/S0090-3019(02)00921-7

[33] Alcalá-Cerra G, Moscote-Salazar LR, Paternina-Caicedo Á, Gutiérrez-Paternina JJ, Niño-Hernández LM, Sabogal-Barrios R (2014) Postoperative bed header position after burr-hole drainage of chronic subdural haematoma: systematic review and meta-analysis of randomised controlled trials. Neurocir Astur Spain 25:99–107. 10.1016/j.neucir.2013.11.002 10.1016/j.neucir.2013.11.002 24657262

[34] Page MJ, McKenzie JE, Bossuyt PM, Boutron I, Hoffmann TC, Mulrow CD et al (2021) The PRISMA 2020 statement: an updated guideline for reporting systematic reviews. BMJ 372:n71. 10.1136/bmj.n71 33782057 10.1136/bmj.n71 PMC8005924

[35] McGuinness LA, Higgins JPT (2021) Risk-of-bias VISualization (robvis): an R package and Shiny web app for visualizing risk-of-bias assessments. Res Synth Methods 12:55–61. 10.1002/jrsm.1411 32336025 10.1002/jrsm.1411

